# MicroRNA Biomarkers for Coronary Artery Disease?

**DOI:** 10.1007/s11883-015-0548-z

**Published:** 2015-10-21

**Authors:** Dorothee Kaudewitz, Anna Zampetaki, Manuel Mayr

**Affiliations:** King’s British Heart Foundation Centre, King’s College London, 125 Coldharbour Lane, London, SE59NU UK

**Keywords:** MicroRNA, Biomarker, Coronary heart disease, Cardiovascular, Myocardial infarction

## Abstract

MicroRNA (miRNA, miR) measurements in patients with coronary heart disease are hampered by the confounding effects of medication commonly used in cardiovascular patients such as statins, antiplatelet drugs, and heparin administration. Statins reduce the circulating levels of liver-derived miR-122. Antiplatelet medication attenuates the release of platelet-derived miRNAs. Heparin inhibits the polymerase chain reactions, in particular the amplification of the exogenous *Caenorhabditis elegans* spike-in control, thereby resulting in an artefactual rise of endogenous miRNAs. As these limitations have not been previously recognised, a reevaluation of the current miRNA literature, in particular of case–control studies in patients with cardiovascular disease or coronary interventions, is required.

## Introduction

Before the completion of the Human Genome Project, the genome was expected to contain at least 100,000 protein-coding genes. DNA sequencing, however, revealed that only about 21,000 such genes can be found within the approximately 3 billion DNA bases. Although approximately 76 % of the human genome is transcribed, less than 3 % encodes for proteins [1]. The remaining 97 % were thought to be ‘junk’ DNA as these sequences are not translated into protein and were not known to encode relevant information with the exception of few non-coding RNAs such as transfer or ribosomal RNAs. This assumption was based on the central dogma of molecular biology postulated by Francis Crick in 1958, which states that all relevant biological information flows unidirectionally from DNA to mRNA to protein [2–4]. As the number of protein-coding genes was unexpectedly low and similar to much simpler organisms, it was not immediately clear how the high degree of human complexity could derive from such a small protein repertoire. Additionally, the proteomes of higher organisms have been shown to be relatively stable, with humans and mice sharing 99 % of their protein-coding genes. Though mechanisms like alternative splicing can increase the variation of the protein repertoire, today, the complexity of the regulatory network of non-coding RNAs, i.e. the control architecture of the system, is thought to be the main source of diversity [5, 6]. This is supported by the fact that while there is only a small increase in the number of protein-coding genes in humans as compared to the nearly 19,000 protein-coding genes in the nematode *Caenorhabditis elegans*, the ratio of non-protein coding to protein-coding sequences is almost 17-fold higher in humans [6, 7]. MicroRNAs (miRNAs) represent one subgroup of non-coding RNAs. More than 2000 miRNAs are encoded in the human genome, but only about 400 miRNAs can be found in *C. elegans* (http://www.mirbase.org/). The miRNA system is also highly evolutionarily conserved: 196 miRNA families are conserved among mammals and 34 miRNA families from *C. elegans* are conserved in humans with few examples of secondary loss and very low levels of nucleotide substitutions to the primary sequence [8, 9]. Epigenetic control mechanisms, including miRNAs that temporarily modulate gene expression, allow the cell to respond quickly to environmental changes as messenger RNA (mRNA) molecules can be targeted for degradation or blocked from translation into proteins [10].

## Biogenesis and Function of miRNAs

miRNAs are one of the largest gene families [11]. Due to their mode of operation, they have the potential to target approximately 60 % of human genes and thereby influence many biological pathways [12, 13]. miRNAs are expressed in a temporal and tissue-specific manner [14], e.g. miR-208a can only be found in cardiomyocytes [15] and the miRNA content in different cells can vary from 1 to more than 30,000 copies [16]. miRNAs play an important role in the regulation of embryonic development as well as in adult life with distinct expression profiles in every cell type at each developmental stage [17]. They show dynamic and site-specific expression patterns during embryogenesis and suppression leads to death during early gestation [18, 19]. The first miRNA was identified in 1993 during the study of *C. elegans* mutants that exhibited abnormal developmental timing. The gene responsible for this phenotype, *lin*-*4*, did not code for a protein but for two small RNAs with complementary sequences to the 3′-untranslated region of the *lin*-*14* mRNA. *Lin*-*4* had been shown previously to negatively regulate the protein level of LIN-14, creating a temporal decrease in LIN-14 during postembryonic development that regulates the execution of stage-specific larval programmes [2, 20, 21]. A similar mechanism of RNA silencing had been known in plants since the beginning of the 1990s [22], but as *lin*-*4* was not detected in other species, this new mechanism was believed to be a process occurring only in nematodes. However, in the year 2000, *let*-*7*, that also coordinates developmental timing in C. *elegans*, was discovered. *Let*-*7* codes for a 21-nucleotide non-coding RNA transcript that negatively regulates the mRNA of *lin*-*41* through complementary Watson-Crick base pairing at the 3′-UTR and therefore influences gene expression in a way similar to *lin*-*4* [19]. Unlike *lin*-*4*, the sequence of *let*-*7* was found to be conserved in a wide range of species including vertebrates, making the biological significance of this finding apparent [4, 23]. Since then, over 25,000 miRNAs have been identified from more than 190 different species including algae, plants, nematodes, protozoa, viruses or vertebrates [4].

**Fig. 1:**
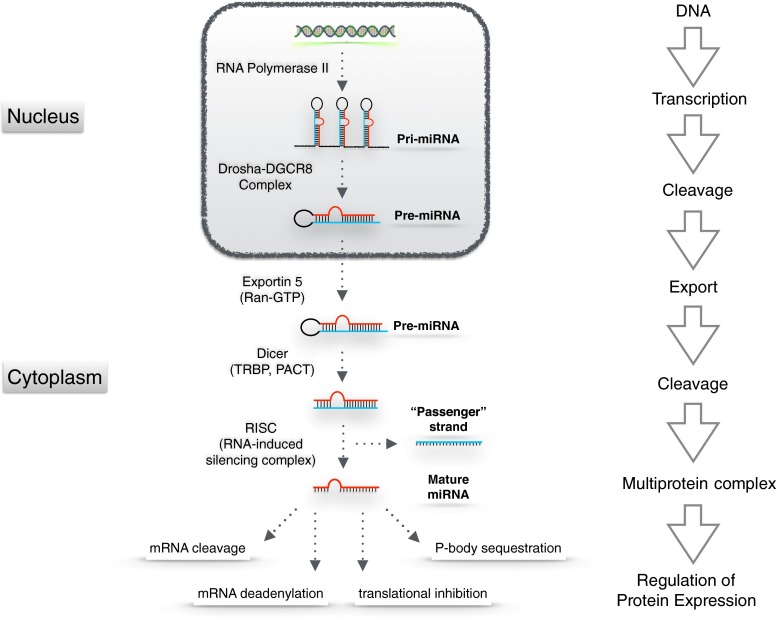
Schematic diagram of miRNA biogenesis and proposed mechanisms for miRNA function. MiRNAs can affect protein expression by inducing an Ago-mediated cleavage of the mRNA, or destabilisation and degradation of mRNA by deadenylation, translational inhibition and sequestration of mRNAs in P-bodies. Adapted from [4, 75, 76]

MiRNA genes are located in different parts of the human genome and hence show a great variation in their transcriptional regulation and expression patterns [24]. The majority of human miRNAs are co-expressed with their host gene within intronic sequences of protein-coding genes, while others are transcribed independent of coding genes [13]. More than 40 % of human miRNAs appear in clusters and are transcribed together, forming a transcript that contains multiple miRNA sequences [25]. After transcription by mainly RNA polymerase II [26], the primary miRNA (pri-miRNA) is processed by the RNase III Drosha, into a stem-loop precursor of ~70 nucleotides, the so called pre-miRNA [27] (Fig. 1). As Drosha by itself cannot bind pri-miRNAs sufficiently, it interacts with the cofactor DGCR8, forming the microprocessor complex [28]. The pre-miRNA is then exported to the cytoplasm in a Ran-GTP dependent manner by Exportin-5 that specifically binds the pre-miRNA [29]. Once it has reached the cytoplasm, the pre-miRNA is cleaved by the RNaseIII Dicer together with the cofactor TRBP [30, 31] and PACT [32] into a duplex, consisting of two miRNA strands [33]. After transcription, the individual miRNAs can be additionally regulated by adenosine deaminases that convert adenosine to inosine and thereby influence the hybridisation of miRNAs to their targets [34]. The RNA duplex is subsequently loaded onto an Argonaute protein, together forming the RNA-induced silencing complex (RISC). After the duplex has been unwound, one of the strands is released and in most cases degraded. The other strand guides the RISC to miRNA response elements (MRE) of the target gene that are complementary to its sequence [4, 8]. MiRNAs mainly target mRNAs, but also have the potential to bind to a wide variety of other molecules including tRNAs or rRNAs [35]. Each miRNA locus produces two mature miRNAs, the ‘guide’ strand with a prevalence of 96–99 % and the ‘passenger’ miRNA* strand [8]. miRNA strand selection depends on different factors including the thermodynamic stability at the ends of the miRNA:miRNA* duplex [36, 37]. Though the miRNA* strand is usually degraded, in some cases, both strands can be functional [37] with either overlapping or different target sites [38]. miRNA*-sequences are likely to have functional relevance in small RNA regulatory networks, as they are highly evolutionary conserved with e.g. more than 40 % of miRNA*-sequences resisting nucleotide divergence across *Drosophila* evolution [39]. Given that both the ‘mature’ and the ‘passenger’ strands can be functional, the miRNA/miRNA* nomenclature has now been retired and instead, the two sequences are referred to as 5-p or 3-p strand of the respective miRNA.

Once they have bound to their target mRNA, miRNAs are able to regulate gene expression in different and sometimes opposite ways depending on factors such as the degree of complementarity with the target mRNA. Near-perfect complementarity of miRNAs and their targets which mainly occurs in plants leads to direct cleavage of the target mRNA [40]. In animals, however, target recognition in most cases does not require perfect complementarity but mainly depends on pairing to the ‘miRNA seed’, the nucleotides 2–8 of the 5′ portion of the miRNA [41]. The short seed match and incomplete base pairing enable the miRNA to target different RNA molecules while a single target gene can contain multiple conserved regions of complementarity [41, 42]. The predominant mechanism by which miRNAs reduce protein output is by triggering deadenylation of the target mRNA, which makes the mRNA more susceptible to degradation [43]. Additionally, miRNAs can inhibit eukaryotic initiation factors [44] or interfere with translational elongation [45]. In some cases, the miRNA response has been reported to switch from inhibition of gene expression to enhancement, thereby e.g. inducing up-regulation of target mRNAs on cell cycle arrest and repressing translation in proliferating cells [4, 46]. These characteristics create a complex regulatory control network that changes in relation to age, developmental or pathophysiological state of the cell, and involves multiple cooperative effects on a large number of targets enabling miRNAs to control various pathways at different levels [47].

## Circulating MiRNAs

The majority of miRNAs are located intracellularly. In 2007, however, miRNAs were found in exosomes, in which they were delivered to other cells allowing gene-based communication between cells [48]. Sequence motifs present in miRNAs can thereby enable specific interaction and loading into exosomes [49]. This transfer of RNA through exosomes might enable local and systemic intercellular exchange of biological information [48]. In the following years, miRNAs were detected in most extracellular biological fluids including serum, plasma, saliva and urine where they showed distinct compositions [50]. MiRNAs can be released into the blood circulation by various mechanisms, including active secretion, apoptosis or necrosis. These miRNAs circulate in different types of vesicles, such as apoptotic bodies, microvesicles (100–1000 nm), exosomes (50–100 nm) and lipoproteins [51]. Many extracellular miRNAs in circulation, however, are also independent of vesicles and are associated with RNA-binding proteins like Argonaute 2 protein, a part of the RNA-induced silencing complex [52]. Human miRNAs isolated from plasma are highly stable in boiling water and resistant to very high or low pH, prolonged room temperature incubation or repeated freeze-thawing [53]. Compared to endogenous plasma miRNAs, synthetic miRNAs are rapidly degraded when added to human plasma unless the RNase activity was inactivated beforehand [54]. Therefore, though miRNAs are susceptible to degradation, circulating miRNAs are resistant to RNase activity as they are secreted in a complex with other molecules such as Argonaute proteins or lipoproteins or in membrane-derived vesicles [53].

Deregulated levels of circulating miRNAs have been linked to different disease states [24]. During cellular stress or pathophysiological conditions such as hypoxia, miRNAs can provide an efficient way of gene regulation to allow the cells to adopt and recover [55]. As they are disease-specifically modulated and easily accessible, circulating miRNAs are potential blood-based biomarkers, useful for diagnostic application e.g. in screening programmes and for monitoring of treatment response or outcome prediction [4, 51]. Based on their biology, circulating miRNAs may have a high level of sensitivity and specificity allowing early and reliable detection of pathological states [47]. Arguably, miRNAs offer some advantages over the most commonly used biomarkers [56]: miRNAs are often expressed in a tissue-, development- or disease-specific manner and the circulating levels of miRNAs are reproducible and consistent among individuals of the same species [51, 57]. Compared to numerous serum proteins, including various processing variants, and posttranslationally modified proteins, there are far fewer known miRNA species, making it possible to obtain a comprehensive profile. In addition, due to their small size and chemical composition, miRNAs are less complex than most other biological molecules and more stable in plasma than mRNAs [57]. Also, miRNAs can be quantified cost-effectively using real-time polymerase chain reaction and a profile can be obtained by next-generation sequencing or microarrays.

## MiRNAs as Biomarkers for Coronary Heart Disease

An example for the potential use of miRNAs as biomarkers is the detection of myocardial infarction, where they might complement the existing biomarkers, such as cardiac troponins. There is still a need for novel biomarkers, as troponins fail to rule out myocardial infarction immediately on admission and are not reliable in certain groups of patients [51, 53]. Another major limitation is their lack of specificity, as unspecific elevation of troponin levels can be caused by non-ischemic conditions such as heart failure and renal disease [58]. MiRNAs that are specifically expressed in the heart muscle, like miR-208a, which is involved in the regulation of myosin heavy chain production during cardiac development [15], have the potential to improve diagnosis of myocardial infarction. In a study with 33 consecutive AMI and 30 non-AMI patients that presented with chest pain, miR-208a remained undetectable in plasma of non-AMI patients including patients with chronic renal failure or trauma, but it was initially detected in 90.9 % of AMI patients and in 100 % of AMI patients within 4 h of the onset of chest pain, even in patients where cardiac troponin I (cTnI) levels were not yet affected [59]. This earlier miRNA peak might be caused by a faster release of miRNAs from damaged cardiomyocytes, as miRNAs are mainly bound to protein complexes in the cytosol while most of the cTnI is bound to myofibrils [60]. An overview of miRNA studies on coronary heart disease and myocardial infarction has been published elsewhere [61, 62]. A major shortcoming of the current literature is the lack of large cohort studies (Table 1). Few prospective studies on coronary heart disease have been published to date [63]. Instead, circulating miRNAs have been measured in numerous small case–control studies without adequate consideration of the effects of comorbidities [64] and medication [61, 62].

**Table 1:** Selected studies on circulating miRNAs and coronary heart disease (CHD)

First author	Description of study population	Study size	No. of miRNAs	Normalisation	Additional correlations
Wang et al. [59]	Patients with AMI vs patients with non-MI CHD vs patients with other CVD	33 vs 16 vs 17	4	*Cel*-*miR*-*39*	cTnI
Chen et al. [77]	Patients with CHD	85	1	*Cel*-*miRs*	IL-1β, endothelium-dependent flow-mediated vasodilation
Jansen et al. [78]	Patients with stable CAD, 6-year follow-up	181	10	*Cel*-*miR*-*39*	Circulating MVs, exosomes, vesicle-free plasma
Huang et al. [79]	Patients with AMI vs healthy controls	I: 178 vs 198 II: 150 vs 150	2	*Cel*-*miR*-*39*	CK, CKMB, cTnI Heparinised patients were excluded
Finn et al. [80]	Patients with significant CHD vs patients with only CHD risk factors vs healthy subjects	21 vs 20 vs 27	8	miR-346	Circulating MPs
Liebetrau et al. [81]	Patients with HOCM, before/after TASH	21	4	*Cel*-*miR*-*39*	hs-cTnT
De Rosa et al. [82]	Patients with stable CAD vs patients with ACS vs patients without CAD	31 vs 19 vs 7	7	*Cel*-*miR*-*39*	hsTnT
Fichtlscherer et al. [68]	Patients with CAD vs healthy controls	I: 36 vs 17 II: 31 vs 14	8	*Cel*-*miR*-*39*	-
Wang et al. [83]	Patients with AMI vs patients with angina pectoris vs control subjects	13 vs 176 vs 127	1	U6	cTnI
Orlemans et al. [84]	Patients with ACS vs controls	106 vs 226	5	RNU6	cTnI, hs-TnT

*CVD* cardiovascular disease, *CAD* coronary artery disease, *ACS* acute coronary syndrome, *HOCM* hypertrophic cardiomyopathy, *TASH* transcoronary ablation of septal hypertrophy, *MV* microvesicles, *MP* microparticles, *CK* creatine kinase, *cTnI* cardiac troponin I, *cTnT* cardiac troponin T

Statins, for example, reduce the circulating levels of the liver-derived miRNA, miR-122 [65•]. Our study by Willeit and Zampetaki et al. [66••] identified circulating platelet miRNAs that are responsive to antiplatelet therapy. In healthy volunteers, prolonged platelet inhibition over 4 weeks affected the levels of plasma miRNAs and resulted in a reduction of several miRNAs, including miR-126 (*P* < 0.001), miR-150 (*P* = 0.003), miR-191 (*P* = 0.004), and miR-223 (*P* = 0.016). Similar results were obtained in patients with symptomatic carotid atherosclerosis (*n* = 33) who were on 75-mg aspirin (ASA) at baseline. After initiation of dual antiplatelet therapy with either dipyridamole or clopidogrel, miRNA changes were observed after 48 h. An effect of ASA on miR-126 plasma levels was also demonstrated by de Boer et al. [67•]. Although the miRNA content of platelets is low compared with other cells, platelets contribute substantially to the circulating miRNA pool. Antiplatelet therapy was a likely confounding factor in previous case–control studies reporting a loss of miRNAs in patients with coronary artery disease [62, 68].

**Fig. 2:**
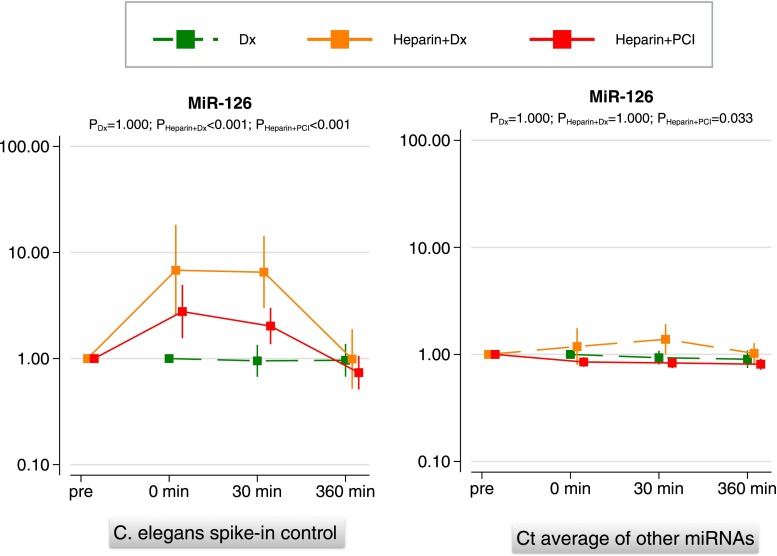
Normalisation of miRNA measurements. Platelet-poor EDTA plasma was collected from patients undergoing percutaneous coronary intervention (PCI, *n* = 20) at four time points: before heparin administration (TP-pre), 5 min after heparin administration but just prior to stent deployment (TP-0 min), and 30 and 360 min after stent deployment (TP, 30 min and TP, 360 min). Additional samples were obtained from patients undergoing cardiac catheterisation for diagnostic purposes (Dx) with (*n* = 7) and without (*n* = 10) heparin administration. Note the discrepancies at TP 0 min and TP 30 min with the conventional normalisation of miRNA measurements using an exogenous *C.elegans* spike-in control (*Cel*-miR *39*, *left panel*) compared to the normalisation using the average cycle threshold (Ct) value of a panel of endogenous miRNAs (*right panel*). Reproduced with permission from [71•]

Similarly, heparin, used in interventional cardiology, is another potentially confounding factor that may influence miRNA measurements due to its known interference with polymerase chain reactions [69, 70]. In the study by Kaudewitz et al. [71•], platelet-poor plasma was obtained from patients undergoing cardiac catheterisation for diagnostic coronary angiography, or for percutaneous coronary intervention, both before and after heparin administration. Heparin had pronounced effects on the assessment of the exogenous *C. elegans* spike-in control (decrease by approx. 3 cycles), which disappeared 6 h after the heparin bolus. Measurements of endogenous miRNAs were less sensitive to heparin medication. Similar findings were reported by Boeckel and colleagues [72•]. Kaudewitz et al. [71•] suggested a potential solution for measuring miRNAs more accurately in heparinized patients: normalisation of individual miRNAs with the average cycle threshold value of all miRNAs provided a suitable alternative to normalisation with exogenous *C. elegans* spike-in control in this setting (Fig. 2). Thus, both the timing of blood sampling relative to heparin dosing and the normalisation procedure are critical for reliable miRNA measurements in patients receiving intravenous heparin. Otherwise, the rise in circulating miRNAs post myocardial infarction can be misinterpreted as novel biomarkers for myocardial injury, whereas the elevation in patients compared to controls may, at least in part, be attributable to the heparin-induced suppression of the exogenous *C. elegans* normalisation control commonly used in miRNA measurements [73].

## Conclusions

**Fig. 3:**
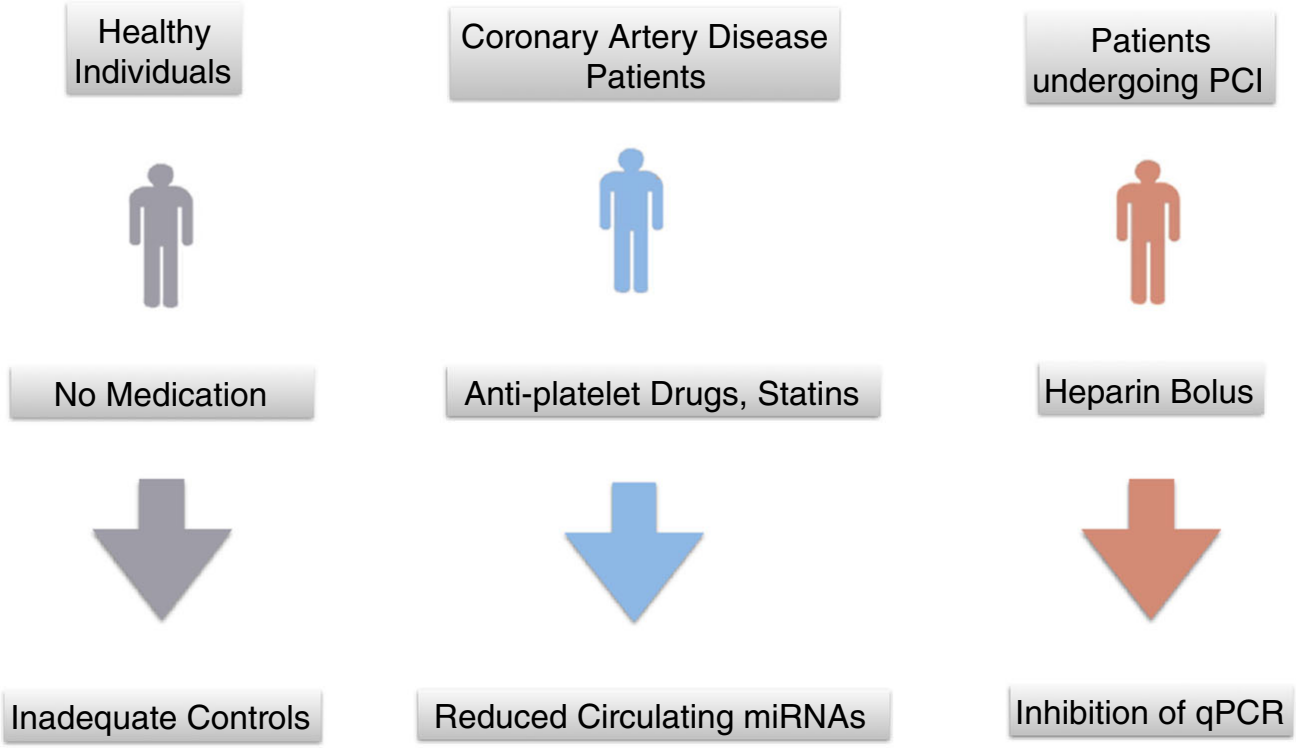
Confounding of miRNA measurements by medication. Antiplatelet medication and statins reduce the endogenous levels of platelet- and liver-derived miRNAs, respectively. Heparin predominantly affects the exogenous spike-in controls commonly used for normalisation. Yet, most case–control studies on miRNA biomarkers for coronary heart disease did not adjust or adequately control for the effects of medication

In summary, medication is an important confounding factor when investigating the relation of circulating miRNAs with coronary heart disease (Fig. 3). Some effects may also apply to measurements in full blood [74]. Future studies will need to address these shortcomings of the early literature on miRNA biomarkers and overcome confounding factors of miRNA measurements to assess the clinical utility of miRNA biomarkers in coronary heart disease.

## Abbreviations

Ago: Argonaute
AMI: acute myocardial infarction
*C.elegans*: *Caenorhabditis elegans*
cTnI: cardiac troponin I
DGCR8: DiGeorge critical region 8
miRNA: microRNA
mRNA: messenger RNA
PACT: protein activator of the interferon-induced protein kinase
Ran-GTP: Ras-related nuclear protein-guanosine triphosphate
RISC: RNA-induced silencing complex
RNase: ribonuclease
TRBP: transactivation response RNA-binding protein
tRNA: transfer RNA
UTR: untranslated region

## References

[1] Pennisi E (2012). Genomics. ENCODE project writes eulogy for junk DNA. Science.

[2] Dogini DB, Pascoal VD, Avansini SH, Vieira AS, Pereira TC, Lopes-Cendes I (2014). The new world of RNAs. Genet Mol Biol.

[3] Crick F (1970). Central dogma of molecular biology. Nature.

[4] Lawrie CH. MicroRNAs: a brief introduction. In: MicroRNAs in Medicine. John Wiley & Sons, Inc.; 2013.1–24.

[5] Mattick JS (2001). Non-coding RNAs: the architects of eukaryotic complexity. EMBO Rep.

[6] Edelstein LC, Nagalla S, Bray PF. MicroRNAs in platelet production and activation. In: MicroRNAs in Medicine. John Wiley & Sons, Inc.; 2013. 101–116.

[7] Shabalina SA, Spiridonov NA (2004). The mammalian transcriptome and the function of non-coding DNA sequences. Genome Biol.

[8] Ha M, Kim VN (2014). Regulation of microRNA biogenesis. Nat Rev Mol Cell Biol.

[9] Wheeler BM, Heimberg AM, Moy VN, Sperling EA, Holstein TW, Heber S (2009). The deep evolution of metazoan microRNAs. Evol Dev.

[10] Aghabozorg Afjeh SS, Ghaderian SM (2013). The role of microRNAs in cardiovascular disease. Int J Mol Cell Med.

[11] Kim VN (2005). MicroRNA biogenesis: coordinated cropping and dicing. Nat Rev Mol Cell Biol.

[12] Friedman RC, Farh KK, Burge CB, Bartel DP (2009). Most mammalian mRNAs are conserved targets of microRNAs. Genome Res.

[13] Siddeek B, Inoubli L, Lakhdari N, Rachel PB, Fussell KC, Schneider S (2014). MicroRNAs as potential biomarkers in diseases and toxicology. Mutat Res Genet Toxicol Environ Mutagen.

[14] Wienholds E, Kloosterman WP, Miska E, Alvarez-Saavedra E, Berezikov E, de Bruijn E (2005). MicroRNA expression in zebrafish embryonic development. Science.

[15] Cordes KR, Srivastava D (2009). MicroRNA regulation of cardiovascular development. Circ Res.

[16] Chen C, Ridzon DA, Broomer AJ, Zhou Z, Lee DH, Nguyen JT (2005). Real-time quantification of microRNAs by stem-loop RT-PCR. Nucleic Acids Res.

[17] Bostjancic E, Glavac D (2014). miRNome in myocardial infarction: future directions and perspective. World J Cardiol.

[18] Aboobaker AA, Tomancak P, Patel N, Rubin GM, Lai EC (2005). Drosophila microRNAs exhibit diverse spatial expression patterns during embryonic development. Proc Natl Acad Sci U S A.

[19] Reinhart BJ, Slack FJ, Basson M, Pasquinelli AE, Bettinger JC, Rougvie AE (2000). The 21-nucleotide let-7 RNA regulates developmental timing in Caenorhabditis elegans. Nature.

[20] Lee RC, Feinbaum RL, Ambros V (1993). The C. elegans heterochronic gene lin-4 encodes small RNAs with antisense complementarity to lin-14. Cell.

[21] Wightman B, Ha I, Ruvkun G (1993). Posttranscriptional regulation of the heterochronic gene lin-14 by lin-4 mediates temporal pattern formation in C. elegans. Cell.

[22] Napoli C, Lemieux C, Jorgensen R (1990). Introduction of a chimeric chalcone synthase gene into petunia results in reversible co-suppression of homologous genes in trans. Plant Cell.

[23] Pasquinelli AE, Reinhart BJ, Slack F, Martindale MQ, Kuroda MI, Maller B (2000). Conservation of the sequence and temporal expression of let-7 heterochronic regulatory RNA. Nature.

[24] Erson-Bensan AE. miRNomics: microRNA biology and computational analysis: introduction to microRNAs in biological systems. 2014;1107. 10.1007/978-1-62703-748-8_1 24272428

[25] Altuvia Y, Landgraf P, Lithwick G, Elefant N, Pfeffer S, Aravin A (2005). Clustering and conservation patterns of human microRNAs. Nucleic Acids Res.

[26] Lee Y, Kim M, Han J, Yeom KH, Lee S, Baek SH (2004). MicroRNA genes are transcribed by RNA polymerase II. EMBO J.

[27] Lee Y, Ahn C, Han J, Choi H, Kim J, Yim J (2003). The nuclear RNase III Drosha initiates microRNA processing. Nature.

[28] Yeom KH, Lee Y, Han J, Suh MR, Kim VN (2006). Characterization of DGCR8/Pasha, the essential cofactor for Drosha in primary miRNA processing. Nucleic Acids Res.

[29] Yi R, Qin Y, Macara IG, Cullen BR (2003). Exportin-5 mediates the nuclear export of pre-microRNAs and short hairpin RNAs. Genes Dev.

[30] Haase AD, Jaskiewicz L, Zhang H, Laine S, Sack R, Gatignol A (2005). TRBP, a regulator of cellular PKR and HIV-1 virus expression, interacts with Dicer and functions in RNA silencing. EMBO Rep.

[31] Chendrimada TP, Gregory RI, Kumaraswamy E, Norman J, Cooch N, Nishikura K (2005). TRBP recruits the Dicer complex to Ago2 for microRNA processing and gene silencing. Nature.

[32] Lee Y, Hur I, Park SY, Kim YK, Suh MR, Kim VN (2006). The role of PACT in the RNA silencing pathway. EMBO J.

[33] Grishok A, Pasquinelli AE, Conte D, Li N, Parrish S, Ha I (2001). Genes and mechanisms related to RNA interference regulate expression of the small temporal RNAs that control C. elegans developmental timing. Cell.

[34] Kawahara Y, Zinshteyn B, Sethupathy P, Iizasa H, Hatzigeorgiou AG, Nishikura K (2007). Redirection of silencing targets by adenosine-to-inosine editing of miRNAs. Science.

[35] Helwak A, Kudla G, Dudnakova T, Tollervey D (2013). Mapping the human miRNA interactome by CLASH reveals frequent noncanonical binding. Cell.

[36] Khvorova A, Reynolds A, Jayasena SD (2003). Functional siRNAs and miRNAs exhibit strand bias. Cell.

[37] Schwarz DS, Hutvagner G, Du T, Xu Z, Aronin N, Zamore PD (2003). Asymmetry in the assembly of the RNAi enzyme complex. Cell.

[38] Marco A, Macpherson JI, Ronshaugen M, Griffiths-Jones S (2012). MicroRNAs from the same precursor have different targeting properties. Silence.

[39] Okamura K, Phillips MD, Tyler DM, Duan H, Chou YT, Lai EC (2008). The regulatory activity of microRNA* species has substantial influence on microRNA and 3′ UTR evolution. Nat Struct Mol Biol.

[40] Rhoades MW, Reinhart BJ, Lim LP, Burge CB, Bartel B, Bartel DP (2002). Prediction of plant microRNA targets. Cell.

[41] Lewis BP, Shih IH, Jones-Rhoades MW, Bartel DP, Burge CB (2003). Prediction of mammalian microRNA targets. Cell.

[42] Nazari-Jahantigh M, Egea V, Schober A, Weber C. MicroRNA-specific regulatory mechanisms in atherosclerosis. J Mol Cell Cardiol. 2014. 10.1016/j.yjmcc.2014.10.021 25450610

[43] Guo H, Ingolia NT, Weissman JS, Bartel DP (2010). Mammalian microRNAs predominantly act to decrease target mRNA levels. Nature.

[44] Humphreys DT, Westman BJ, Martin DI, Preiss T (2005). MicroRNAs control translation initiation by inhibiting eukaryotic initiation factor 4E/cap and poly(A) tail function. Proc Natl Acad Sci U S A.

[45] Olsen PH, Ambros V (1999). The lin-4 regulatory RNA controls developmental timing in *Caenorhabditis elegans* by blocking LIN-14 protein synthesis after the initiation of translation. Dev Biol.

[46] Vasudevan S, Tong Y, Steitz JA (2007). Switching from repression to activation: microRNAs can up-regulate translation. Science.

[47] Condorelli G, Latronico MV, Cavarretta E (2014). MicroRNAs in cardiovascular diseases: current knowledge and the road ahead. J Am Coll Cardiol.

[48] Valadi H, Ekstrom K, Bossios A, Sjostrand M, Lee JJ, Lotvall JO (2007). Exosome-mediated transfer of mRNAs and microRNAs is a novel mechanism of genetic exchange between cells. Nat Cell Biol.

[49] Villarroya-Beltri C, Gutierrez-Vazquez C, Sanchez-Cabo F, Perez-Hernandez D, Vazquez J, Martin-Cofreces N (2013). Sumoylated hnRNPA2B1 controls the sorting of miRNAs into exosomes through binding to specific motifs. Nat Commun.

[50] Weber JA, Baxter DH, Zhang S, Huang DY, Huang KH, Lee MJ (2010). The microRNA spectrum in 12 body fluids. Clin Chem.

[51] Schwarzenbach H, Pantel K. Circulating microRNAs as non-invasive biomarkers. In: MicroRNAs in Medicine. John Wiley & Sons, Inc.; 2013. 567–588.

[52] Turchinovich A, Weiz L, Langheinz A, Burwinkel B (2011). Characterization of extracellular circulating microRNA. Nucleic Acids Res.

[53] Sayed AS, Xia K, Yang TL, Peng J (2013). Circulating microRNAs: a potential role in diagnosis and prognosis of acute myocardial infarction. Dis Markers.

[54] Tsui NB, Ng EK, Lo YM (2002). Stability of endogenous and added RNA in blood specimens, serum, and plasma. Clin Chem.

[55] Hata A (2013). Functions of microRNAs in cardiovascular biology and disease. Annu Rev Physiol.

[56] Baulcombe D. Foreword. In: MicroRNAs in Medicine. John Wiley & Sons, Inc.; 2013. i-xviii.

[57] Wang Z: Circulating miRNAs as biomarkers for cardiac disease. In: MicroRNAs and cardiovascular disease 2010: 121-126.

[58] Rawal S, Manning P, Katare R (2014). Cardiovascular microRNAs: as modulators and diagnostic biomarkers of diabetic heart disease. Cardiovasc Diabetol.

[59] Wang GK, Zhu JQ, Zhang JT, Li Q, Li Y, He J (2010). Circulating microRNA: a novel potential biomarker for early diagnosis of acute myocardial infarction in humans. Eur Heart J.

[60] Creemers EE, Tijsen AJ, Pinto YM (2012). Circulating microRNAs: novel biomarkers and extracellular communicators in cardiovascular disease?. Circ Res.

[61] Economou EK, Oikonomou E, Siasos G, Papageorgiou N, Tsalamandris S, Mourouzis K (2015). The role of microRNAs in coronary artery disease: from pathophysiology to diagnosis and treatment. Atherosclerosis.

[62] Mayr M, Zampetaki A, Willeit P, Willeit J, Kiechl S (2013). MicroRNAs within the continuum of postgenomics biomarker discovery. Arterioscler Thromb Vasc Biol.

[63] Zampetaki A, Willeit P, Tilling L, Drozdov I, Prokopi M, Renard JM (2012). Prospective study on circulating microRNAs and risk of myocardial infarction. J Am Coll Cardiol.

[64] Zampetaki A, Kiechl S, Drozdov I, Willeit P, Mayr U, Prokopi M (2010). Plasma microRNA profiling reveals loss of endothelial miR-126 and other microRNAs in type 2 diabetes. Circ Res.

[65.•] Gao W, He HW, Wang ZM, Zhao H, Lian XQ, Wang YS (2012). Plasma levels of lipometabolism-related miR-122 and miR-370 are increased in patients with hyperlipidemia and associated with coronary artery disease. Lipids Health Dis.

[66.••] Willeit P, Zampetaki A, Dudek K, Kaudewitz D, King A, Kirkby NS (2013). Circulating microRNAs as novel biomarkers for platelet activation. Circ Res.

[67.•] de Boer HC, van Solingen C, Prins J, Duijs JM, Huisman MV, Rabelink TJ (2013). Aspirin treatment hampers the use of plasma microRNA-126 as a biomarker for the progression of vascular disease. Eur Heart J.

[68] Fichtlscherer S, De Rosa S, Fox H, Schwietz T, Fischer A, Liebetrau C (2010). Circulating microRNAs in patients with coronary artery disease. Circ Res.

[69] Yokota M, Tatsumi N, Nathalang O, Yamada T, Tsuda I (1999). Effects of heparin on polymerase chain reaction for blood white cells. J Clin Lab Anal.

[70] Satsangi J, Jewell DP, Welsh K, Bunce M, Bell JI (1994). Effect of heparin on polymerase chain reaction. Lancet.

[71.•] Kaudewitz D, Lee R, Willeit P, McGregor R, Markus HS, Kiechl S (2013). Impact of intravenous heparin on quantification of circulating microRNAs in patients with coronary artery disease. Thromb Haemost.

[72.•] Boeckel JN, Thomé CE, Leistner D, Zeiher AM, Fichtlscherer S, Dimmeler S. Heparin selectively affects the quantification of microRNAs in human blood samples. Clin Chem. 2013;59:1125–7. **Demonstrates the effect of a heparin bolus on the exogenous C. elegans normalisation control**. 10.1373/clinchem.2012.199505 23613337

[73] Mayr M, Lee R, Kaudewitz D, Zampetaki A, Channon KM (2014). Effects of heparin on temporal microRNA profiles. J Am Coll Cardiol.

[74] Huan T, Rong J, Tanriverdi K, Meng Q, Bhattacharya A, McManus DD (2015). Dissecting the roles of microRNAs in coronary heart disease via integrative genomic analyses. Arterioscler Thromb Vasc Biol.

[75] Marc R. Fabian TRS, and Nahum Sonenberg. Understanding how miRNAs post-transcriptionally regulate gene expression. In: MiRNA regulation of the translational Machinery. Edited by Rhoads RE: Springer; 2010. 6.

[76] Zampetaki A, Mayr M (2015). Sweet dicer: impairment of micro-RNA processing by diabetes. Circ Res.

[77] Chen Z, Wen L, Martin M, Hsu CY, Fang L, Lin FM (2015). Oxidative stress activates endothelial innate immunity via sterol regulatory element binding protein 2 (SREBP2) transactivation of microRNA-92a. Circulation.

[78] Jansen F, Yang X, Proebsting S, Hoelscher M, Przybilla D, Baumann K (2014). MicroRNA expression in circulating microvesicles predicts cardiovascular events in patients with coronary artery disease. J Am Heart Assoc.

[79] Huang S, Chen M, Li L, He M, Hu D, Zhang X (2014). Circulating microRNAs and the occurrence of acute myocardial infarction in Chinese populations. Circ Cardiovasc Genet.

[80] Finn NA, Eapen D, Manocha P, Al Kassem H, Lassegue B, Ghasemzadeh N (2013). Coronary heart disease alters intercellular communication by modifying microparticle-mediated microRNA transport. FEBS Lett.

[81] Liebetrau C, Mollmann H, Dorr O, Szardien S, Troidl C, Willmer M (2013). Release kinetics of circulating muscle-enriched microRNAs in patients undergoing transcoronary ablation of septal hypertrophy. J Am Coll Cardiol.

[82] De Rosa S, Fichtlscherer S, Lehmann R, Assmus B, Dimmeler S, Zeiher AM (2011). Transcoronary concentration gradients of circulating microRNAs. Circulation.

[83] Wang F, Long G, Zhao C, Li H, Chaugai S, Wang Y (2013). Plasma microRNA-133a is a new marker for both acute myocardial infarction and underlying coronary artery stenosis. J Transl Med.

[84] Oerlemans MI, Mosterd A, Dekker MS, de Vrey EA, van Mil A, Pasterkamp G (2012). Early assessment of acute coronary syndromes in the emergency department: the potential diagnostic value of circulating microRNAs. EMBO Mol Med.

